# Communism in Medicine

**Published:** 1885-08

**Authors:** 


### Medical ★ Journal,

PUBLISHED MONTHLY AT

AUSTIN, TEXAS.

F. E. DANIEL, M. D., EDITOR AND PUBLISHER,

Subscription Price: Two Dollars per Annum in Advance.

Papers for publication in this Journal should be in the hands of the Editor by the 10th of the month preceding the month in which it is wished to appear; they must be original,--never having appeared in print elsewhere, and must be contributed exclusively to this Journal.

Contributions to the department of Original Articles are cordially invited from the profession of Texas, especially. What is most desired is Clinical reports of cases, essays and short practical articles on any medical topic ; also solicited reports of proceedings of societies, clubs, etc., and items of medical news of general interest to the profession, such as notices of appointments of physicians, removals, changes, marriages, deaths, etc.

### EDITORIAL.

### COMMUNISM IN MEDICINE.

In a new country like America, and under a Republican form of government, where the idea of liberty is carried to an extreme limit, it is to be expected that the profession of medicine would be, normally, of a heterogeneous nature. Especially is this true when, in addition to the vain boast that "this is a free country" and every man has a right to do as he pleases,—practice medicine included, without regard to qualification, education, preparation or authorization, (and we may say, also, without regard to consequences,)—we have the deplorable results of competition in medical colleges, manifested in floods of half educated young "doctors" turned loose upon the community, armed with those legal death-warrants, improperly called "diplomas." That a most heterogeneous medical profession did exist throughout these United States, and does now exist to a great extent, no one will deny; and what was once proudly called a learned profession, a science, was fast degenerating into a trade,—a promiscous scramble after the almighty American dollar,—a business, the sole aim of which seemed to be to break down competition by fair means or by foul,—and to fill the pocket.

A few good men, alarmed at the tendency, and the degradation of medicine, made a stand for its salvation, as gallant as stood Leonidas at Thermopylæ, and fought against odds as overwhelming. They rallied a few followers; organization was effected; the subject of a higher education was discussed—agitated,—the profession was aroused to a sense of the danger; steps were taken in some states to restrict the practice under certain penalties; journalism lent its aid, and for a time the downward career of medicine was checked. Then that grand conception originated in the brain of that grand old physician and man N. S. DAVIS,— a National Medical Association! and a new era dawned. These men strove for the elevation and purication of the profession, and advocated higher education, and a higher standard of morals. The Code of Ethics of the American Medical Association was adopted, and became at once the anchor and the compass of Organized National Medicine. Hope dawned that Fair Medicine would once again be installed in her rightful place, the peerless Queen of the Sciences!

But, alas! for the depravity of human nature! A conflict arose in the breast of certain members, a conflict between the moral principle and the greed of gain; and they said in their hearts, "Those restrictions are galling, they interfere, too, with my money making schemes, all is grist that comes to my mill, and a fee from a homœopathic consultation is as good as any fee; we will assert our American independence, and we will do as we please! to the devil with your code, and your fine-spun notions of etiquette and morals; we will consult with whomsoever will bring us a patient and a fee." But for very shame they could not declare their real motive; a pretext was sought, which would justify them, they thought, in the eyes of the world, and enlist sympathy, at the same time, and they said:

"Your code will not let us exercise our calling in the interests of humanity, unless the consultant has a diploma."

On that pretext they left the ranks of organized, rational medicine, while others never came in to it. They construed the code to accommodate their wishes, and made it a pretext, left the Association and joined hands with the eclectic and the homœopath. Then the doors of the American Medical Association were closed behind them. They have since knocked for admittance, but in no spirit of penitence, but because it seemed *interest* could be best promoted by a nominal connection, even, with the organization. They were not admitted; they had voluntarily withdrawn from fellowship with ethical medicine, and defying the code, had placed themselves in antagonism to its spirit.

The American Medical Association extended an invitation in the name of the profession of America to the International Medical Congress to meet here in 1887. The invitation was accepted; then these gentlemen saw their mistake, and realized the value of membership in the only national medical organization in America. The rest is too well known to repeat here. Those not in affiliation with regular medicine are not to be permitted to take part in the Congress, and behold, these self disfranchised men would prevent its meeting!

Meantime the leaders of general medicine in America, still pacific, and with that patience which says, "If a man have one hundred sheep and one of them be gone astray, is it not better to leave the ninety and the nine and seek the one that is gone astray?" called a council of the faithful and said: "These men, our brothers, use us spitefully; they complain that the code interferes with the dictates of humanity; that they are honor-bound to respond to the cry of distress, even though a homœopath be in the case." Let us consider together their grievances and see if we cannot reclaim to their allegiance—"those that be gone astray."

Accordingly a commission was called of wise men, learned, liberal, illustrious men of the nation, to interpret the meaning of the clause in the code to which these gentlemen objected. The matter was well considered, and the following resolution

(among others) was adopted as expressing the true import and meaning of the clause referred to :

"*Resolved*, That there is no provision in the National Code of Medical Ethics in any wise inconsistent with the broadest dictates of humanity, and that the article of the Code which relates to consultations cannot be correctly interpreted as interdicting, under any circumstances, the rendering of professional services whenever there is pressing or immediate need of them. On the contrary, to meet the emergencies occasioned by disease or accident, and to give the helping hand to the distressed without unnecessary delay, is a duty fully enjoined on every member of the profession, both by the letter and the spirit of the entire code."

Thus is the last plank knocked out of the platform on which the seceeders had ostensibly taken their stand; and on this interpretation, which is official, they are, one and all, *honor bound*, in our opinion, if they claim to be honest and consistent in what they have given as the reason for their course, *to return at once to the fold of the Association and to the ranks of legitimate medicine.* They should be *convinced*, and acknowledge their error, or forever *stand convicted* of resorting to the shallowest subterfuge to cover their real and unworthy motive, which seems to be the exercise of privileges whereby money can be made—by practices in violation of the spirit and letter of the code, and inconsistent with the dignity and character of the true physician—*the consultation with quacks and irregulars*!

There is a phase in human nature, which every nation, in every time, has recognized. It is that desire to pull down those who climb higher than we; to deprive ones neighbor of that which one cannot himself have. A phase best expressed by Æsop in the fable of the "Dog in the Manger." The communistic spirit in France is but in exagerated and intensified expression of it. It is that spirit which would uproot society; trusting in the confusion to find profit, and having nothing to lose, and all to gain, they would "let slip the dogs of war and cry havoc." 'Tis the spirit of the *sans culottes* who would destroy the aristocracy because they cannot be arristocrats—level all distinctions, and bring all to their low plane, instead, of by the evercise of virtue, climb to higher spheres.

That spirit has seized upon the profession of medicine in America in certain quarters ; and the effort to break down organized medicine, and to negative the fruits of years of labor of love,in trying to purify,elevate and ennoble the practice, and to restore it to what our fathers made it—a SCIENCE, and an *honorable profession ;* to level all distinctions in medicine, to drag the code of ethics to the guillotine, to raze the very temple of Esculapius and set up one to Mammon in its stead, is but the outgrowth of that revolutionary movement which lead first to the disruption of organized medicine in New York. It aims at the extinction of medicine as a science, and would break down all distinctions and barriers.

The latest phase of it is recognized in the recent movement in the northeast, by men learned, and heretofore considered highly honorable. They were not permitted to conduct the affairs of the International Congress, in the face of authority invoked by themselves, and behold, they will not permit others to do it; but, rather than see reputable medicine maintain its character and dignity in the eyes of the world, they will break up the whole Congress, and, if possible, prevent its meeting. Is not that the spirit of the mastif who could not eat the corn, yet would not let the ox have it?

The communist is at work ; the axe has been laid to the tree of medical science, and its temples given to the torch, while its votaries would be chained and dragged to the guilotine in the blind fury of these medical iconoclasts! It behooves the members of the Hippocratic school to be firm, and to act with discretion. We are on the eve of events which, if unchecked, will bring woful consequences, and leave a chaos where order and peace should reign.

